# Sarcopenic obesity and cachexia as nutritional risk phenotypes and histology-specific outcomes after intracranial tumor resection: a histology-stratified NSQIP analysis of 27,057 cases

**DOI:** 10.1007/s10143-026-04450-3

**Published:** 2026-08-18

**Authors:** Caleigh S. Roach, Belen Wertheimer, Khushi H. Shah, Victor M. Lu, Ashish H. Shah, Ricardo J. Komotar

**Affiliations:** 1 Department of Neurological Surgery, University of Miami Miller School of Medicine, 1295 NW 14th Street, Suite 1700, Miami, FL 33125 USA; 2 Department of Orthopaedic Surgery, University of Miami Miller School of Medicine, Miami, FL USA

**Keywords:** Cranial tumor surgery, Nutritional phenotype, Sarcopenic obesity, Cachexia, Failure to rescue, Inverse probability of treatment weighting

## Abstract

Preoperative risk stratification in neurosurgery typically uses body mass index (BMI), albumin, or frailty as single-marker predictors. We assessed whether a composite phenotype predicts outcomes after cranial neuro-oncologic surgery and whether effects differ by tumor histology. A retrospective analysis of 27,057 patients undergoing craniotomy for meningioma, malignant glioma, or brain metastasis was performed using the American College of Surgeons National Surgical Quality Improvement Program database (2016–2023). The composite phenotype incorporated BMI and serum albumin: adequately nourished, obese-replete, sarcopenic-obese, and cachectic-malnourished. Outcomes included major 30-day morbidity, 30-day mortality, failure to rescue (FTR), and non-home discharge. Associations were estimated using inverse probability of treatment weighting (IPTW), histology-stratified analyses, and interaction testing. Compared with adequately nourished patients, sarcopenic-obese and cachectic-malnourished phenotypes conferred elevated odds of major 30-day morbidity (IPTW odds ratio [OR] 1.47 and 1.60, respectively) and 30-day mortality (OR 2.28 and 1.96). Obese-replete showed no mortality increase (OR 1.06) and reduced FTR (OR 0.76), most pronounced in the metastasis cohort (OR 0.58). Phenotype effects differed by histology for mortality (interaction *p* = 0.044) and non-home discharge (*p* = 0.009), with sarcopenic obesity conferring a 3.61-fold mortality increase among meningioma patients. The composite phenotype achieved discrimination comparable to but not exceeding serum albumin alone (area under the curve [AUC] 0.75 vs. 0.76 for mortality). A biomarker-defined nutritional phenotype separated distinct high-risk subgroups but did not improve discrimination beyond serum albumin alone, with histology-specific effects. These findings support histology-aware nutritional phenotyping for preoperative risk communication and hypothesis generation rather than superior prediction.

**Supplementary information:**

The online version contains supplementary material available at 10.1007/s10143-026-04450-3.

## Introduction

Postoperative complications remain disproportionately prevalent in intracranial tumor resection (ITR), with major 30-day morbidity affecting 10–15% of patients undergoing cranial neuro-oncologic surgery [1–3], and 30-day mortality approaching 4–5% in the metastatic disease setting [4, 5]. Precise preoperative risk stratification is therefore essential for patient selection, prehabilitation, informed consent, and perioperative planning in neuro-oncology.

Body composition, quantified by body mass index (BMI), exhibits a nonlinear relationship with surgical outcomes: both underweight and severe obesity confer elevated risk, whereas moderate obesity may exert paradoxical protective effects depending on nutritional adequacy [6]. Nutritional status, as captured by serum albumin, has predicted mortality and major morbidity since the foundational work of Gibbs and colleagues [7], integrating chronic intake and systemic inflammation [8]. Frailty, conceptualized as diminished baseline physiologic reserve, constitutes a third domain, and has been operationalized using indices such as the Modified Frailty Index-5 (mFI-5) [9] and the Risk Analysis Index (RAI) [10], with the latter showing superior discriminative performance in neurosurgical analyses [11–13].

Despite oncologic and geriatric consensus on sarcopenic obesity and cachexia [14, 15], and identification as principal targets for risk stratification, these markers have seldom been studied together as an integrated phenotype in cranial neuro-oncologic surgery. Prior analyses [1, 4, 13] have also largely pooled cranial tumor histologies or restricted to one group, yet meningioma, malignant glioma, and metastasis differ substantially in tumor biology, extent-of-resection goals, systemic disease burden, adjuvant therapy timelines, and perioperative vulnerability [16–19]. Whether a phenotype defined a priori captures risk beyond that of single markers, and whether phenotype effects vary within neuro-oncology, is unknown [20].

We therefore performed a histology-stratified analysis of ITR outcomes to operationalize a composite nutritional phenotype incorporating BMI and serum albumin, characterize its distribution across meningioma, malignant glioma, and metastasis cohorts, estimate its association with major 30-day morbidity and mortality, and evaluate the sensitivity of phenotype-outcome associations to alternative frailty operationalizations.

## Materials and methods

### Study design and data source

We performed a retrospective cohort analysis of the American College of Surgeons National Surgical Quality Improvement Program (ACS-NSQIP) Participant Use File from 2016 to 2023 [21]. Because the data are de-identified and publicly available under a participant use file agreement, this study was deemed exempt from review by the Institutional Review Board. Reporting follows the Strengthening the Reporting of Observational Studies in Epidemiology (STROBE) guidelines [22]. This study was performed in line with the principles of the Declaration of Helsinki. The study was deemed exempt from review by the University of Miami Institutional Review Board, which waived the requirement for informed consent because the analysis used de-identified data available under a Participant Use File agreement with the American College of Surgeons. Clinical trial number: not applicable.

### Cohort selection

Adult patients (≥ 18 years) undergoing ITR were identified using Current Procedural Terminology (CPT) codes for craniectomy or craniotomy for tumor and skull base tumor approaches. Cases were excluded for non-neurosurgical specialty, unassignable histologic category, operative time below 60 min, or missing both serum albumin and BMI. Histology was classified into three mutually exclusive cohorts: meningioma, malignant glioma, and brain metastasis. The complete CPT codes used for cohort identification and the algorithm used to assign histology from postoperative ICD-10 diagnosis codes, with CPT as a fallback, are detailed in the Supplementary Methods and Supplementary Table 4.

### Composite nutritional phenotype

The primary exposure was a composite nutritional phenotype derived from BMI and serum albumin, grounded in consensus definitions of sarcopenic obesity and cachexia [14, 15]. BMI (kg/m^2^) was categorized per World Health Organization standards [23]. Serum albumin was dichotomized at 3.5 g/dL based on the Gibbs threshold for clinically meaningful hypoalbuminemia [7]. The phenotype was operationalized into four mutually exclusive categories. These categories are administrative surrogates constructed from body mass index and serum albumin rather than validated clinical diagnoses; because ACS-NSQIP does not capture cross-sectional muscle mass, the sarcopenic-obese and cachectic-malnourished labels denote biomarker-defined risk phenotypes and not imaging- or criterion-confirmed sarcopenia or cachexia. Adequately nourished (reference) was defined as BMI 18.5–29.9 with albumin ≥ 3.5 g/dL. Obese-replete was defined as BMI ≥ 30.0 with albumin ≥ 3.5 g/dL. Sarcopenic obesity was defined as BMI ≥ 30.0 with albumin < 3.5 g/dL, consistent with the European Society for Clinical Nutrition and Metabolism and the European Association for the Study of Obesity (ESPEN/EASO) consensus [15]. Cachectic-malnourished was defined as BMI < 18.5 regardless of albumin, BMI 18.5–29.9 with albumin < 3.5 g/dL, or documented preoperative weight loss greater than 10% of body weight within 6 months in patients with BMI < 30.0 [14].

### Covariates

Covariates included demographics (age, sex, race/ethnicity), frailty (RAI-rev [10] categorized into four tiers), and comorbidities, including American Society of Anesthesiologists (ASA) classification, chronic preoperative corticosteroid use [4], and standard cardiovascular and metabolic conditions (full list in Table 1 footnote). To prevent the phenotype from acting as a proxy for general illness severity, we additionally adjusted for preoperative serum sodium, creatinine, hematocrit, white blood cell count, and international normalized ratio [24]. All covariates were included in the multinomial propensity score model and the multivariable outcome model.

**Table 1:** Unadjusted, multivariable-adjusted, and IPTW-adjusted associations between composite nutritional phenotype and 30-day postoperative outcomes

Outcome / phenotype	n events / n at risk (%)	Unadjusted OR (95% CI), *p*	Adjusted OR (95% CI), *p*	IPTW OR (95% CI), *p*
Major 30-day morbidity				
Adequately nourished	1,727/13,531 (13%)	-	-	-
Obese-replete	1,223/8,084 (15%)	1.22 (1.13-1.32), <0.001***	1.16 (1.06-1.26), <0.001***	1.15 (1.06-1.24), <0.001***
Sarcopenic-obese	388/1,597 (24%)	2.19 (1.94-2.49), <0.001***	1.56 (1.36-1.78), <0.001***	1.47 (1.28-1.68), <0.001***
Cachectic-malnourished	895/3,845 (23%)	2.07 (1.89-2.27), <0.001***	1.58 (1.43-1.75), <0.001***	1.60 (1.46-1.76), <0.001***
30-day mortality				
Adequately nourished	293/13,531 (2.2%)	-	-	-
Obese-replete	172/8,084 (2.1%)	0.98 (0.81-1.19), 0.853	1.15 (0.94-1.40), 0.176	1.06 (0.89-1.26), 0.508
Sarcopenic-obese	110/1,597 (6.9%)	3.34 (2.67-4.19), <0.001***	2.30 (1.81-2.92), <0.001***	2.28 (1.79-2.92), <0.001***
Cachectic-malnourished	296/3,845 (7.7%)	3.77 (3.19-4.45), <0.001***	2.13 (1.78-2.55), <0.001***	1.96 (1.62-2.36), <0.001***
Failure to rescue				
Adequately nourished	165/1,069 (15%)	-	-	-
Obese-replete	92/811 (11%)	0.70 (0.53-0.92), 0.011*	0.81 (0.59-1.10), 0.175	0.76 (0.59-0.99), 0.038*
Sarcopenic-obese	67/259 (26%)	1.91 (1.38-2.64), <0.001***	1.74 (1.23-2.45), 0.002**	1.57 (1.09-2.25), 0.014*
Cachectic-malnourished	138/552 (25%)	1.83 (1.42-2.35), <0.001***	1.33 (0.99-1.80), 0.061†	1.19 (0.90-1.57), 0.217
Non-home discharge				
Adequately nourished	2,710/13,531 (20%)	-	-	-
Obese-replete	1,672/8,084 (21%)	1.04 (0.97-1.11), 0.247	1.06 (0.98-1.14), 0.123	1.05 (0.98-1.12), 0.178
Sarcopenic-obese	563/1,597 (35%)	2.17 (1.95-2.43), <0.001***	1.56 (1.38-1.76), <0.001***	1.55 (1.38-1.74), <0.001***
Cachectic-malnourished	1,278/3,845 (33%)	1.99 (1.84-2.15), <0.001***	1.46 (1.34-1.60), <0.001***	1.50 (1.38-1.63), <0.001***
Prolonged LOS				
Adequately nourished	2,956/13,531 (22%)	-	-	-
Obese-replete	1,860/8,084 (23%)	1.07 (1.00-1.14), 0.047*	1.04 (0.97-1.12), 0.296	1.02 (0.95-1.09), 0.608
Sarcopenic-obese	711/1,597 (45%)	2.87 (2.58-3.19), <0.001***	2.17 (1.93-2.44), <0.001***	2.12 (1.90-2.37), <0.001***
Cachectic-malnourished	1,603/3,845 (42%)	2.56 (2.37-2.76), <0.001***	1.96 (1.80-2.13), <0.001***	1.96 (1.81-2.11), <0.001***
Unplanned readmission				
Adequately nourished	1,444/13,531 (11%)	-	-	-
Obese-replete	924/8,084 (11%)	1.08 (0.99-1.18), 0.084†	1.09 (1.00-1.20), 0.053†	1.10 (1.01-1.20), 0.025*
Sarcopenic-obese	269/1,597 (17%)	1.70 (1.47-1.95), <0.001***	1.40 (1.20-1.62), <0.001***	1.43 (1.24-1.67), <0.001***
Cachectic-malnourished	573/3,845 (15%)	1.47 (1.32-1.63), <0.001***	1.13 (1.01-1.26), 0.030*	1.20 (1.08-1.34), 0.001**
Unplanned reoperation				
Adequately nourished	589/13,531 (4.4%)	-	-	-
Obese-replete	443/8,084 (5.5%)	1.27 (1.12-1.45), <0.001***	1.21 (1.06-1.39), 0.004**	1.24 (1.09-1.41), <0.001***
Sarcopenic-obese	110/1,597 (6.9%)	1.63 (1.32-2.01), <0.001***	1.43 (1.15-1.78), 0.001**	1.44 (1.16-1.80), 0.001**
Cachectic-malnourished	239/3,845 (6.2%)	1.46 (1.25-1.70), <0.001***	1.30 (1.10-1.54), 0.002**	1.33 (1.13-1.56), <0.001***

Adjusted models include age, age squared, sex, race/ethnicity, tumor histology, supratentorial vs. infratentorial location, RAI score, ASA classification ≥ 3, hypertension, diabetes, COPD, congestive heart failure, dialysis, disseminated cancer, smoking, functional dependence, chronic steroid use, emergent presentation, inpatient setting, log-transformed operative time, surgical year era (2020–2023 vs. 2016–2019), and preoperative serum sodium, creatinine, white blood cell count, hematocrit, and INR. IPTW models used stabilized weights from a multinomial propensity score model for the four-level phenotype, truncated at the 1 st and 99th percentiles. Failure to rescue analyzed only among patients experiencing a qualifying major complication (n = 2,691). *p < 0.05; **p < 0.01; ***p < 0.001; † p < 0.10

^1^*or* =odds ratio, *ci* confidence interval, *iptw* inverse probability of treatment weighting, *los* length of stay

### Outcomes

Co-primary outcomes were major 30-day morbidity and 30-day mortality. Major morbidity included surgical site infection, pneumonia, unplanned reintubation, ventilator dependence beyond 48 h, pulmonary embolism, deep venous thrombosis, cardiac arrest, myocardial infarction, cerebrovascular accident, acute renal failure or progressive renal insufficiency, sepsis or septic shock, bleeding requiring transfusion, wound dehiscence, or urinary tract infection. Secondary outcomes were FTR, defined as 30-day mortality following a major postoperative complication [7, 25], and non-home discharge (rehabilitation, skilled nursing, acute care transfer, or hospice). FTR-qualifying complications were prespecified as unplanned reintubation, ventilator dependence beyond 48 h, pulmonary embolism, deep venous thrombosis, cardiac arrest, myocardial infarction, cerebrovascular accident, acute renal failure or progressive renal insufficiency, sepsis or septic shock, or organ-space surgical site infection; pneumonia, superficial and deep surgical site infection, urinary tract infection, bleeding requiring transfusion, and wound dehiscence were not counted as FTR-qualifying, yielding 2,691 patients at risk versus the 4,233 patients with major morbidity. Tertiary outcomes were prolonged length of stay (> 75th percentile within histologic stratum [LOS]), unplanned 30-day readmission, and unplanned reoperation.

### Statistical analysis

Descriptive statistics compared baseline characteristics using one-way analysis of variance (ANOVA), Kruskal-Wallis, chi-squared, or Fisher exact tests as appropriate. Covariate balance was assessed using standardized mean differences (SMDs), with values > 0.10 considered meaningful. Phenotype-outcome associations were estimated using unadjusted, multivariable-adjusted, and inverse probability of treatment weighting (IPTW) logistic regression with stabilized weights from a multinomial propensity model; balance was reassessed after weighting. Effect modification by tumor histology was evaluated using phenotype-histology interaction terms with likelihood ratio tests and stratified estimates. Incremental discrimination was assessed by comparing area under the receiver operating characteristic curve (AUC) across nested models containing the phenotype, individual markers (BMI, albumin, RAI), and combined continuous/discretized specifications. Primary analyses used complete BMI and albumin cases. Sensitivity analyses included multiple imputation with Rubin’s rules, substitution of RAI with mFI-5 and ACS-NSQIP universal surgical risk calculator-derived MORTPROB [26], and RAI removal. Secondary and interaction analyses were not adjusted for multiple comparisons; the reported p values for these analyses, including the histology interaction terms, are nominal and interpreted as exploratory. Analyses were performed in Python 3.12 and R 4.4, with two-sided α = 0.05.

## Results

###  Cohort and nutritional phenotype distribution

Of 433,543 adult cranial procedures in the ACS-NSQIP Participant Use File from 2016 to 2023, 47,019 cases met the inclusion criteria for ITR across the three primary histologic strata. Of these, 27,057 (58%) had complete data for both BMI and serum albumin and were eligible for the primary phenotype-based analysis (Supplementary Fig. 1). Meningioma accounted for 6,592 cases (24%), malignant glioma for 11,873 (44%), and brain metastasis for 8,592 (32%). Median age was 61 years (IQR 50–69), 51% were female, and 63% were White non-Hispanic; the cohort skewed toward the robust RAI tier (78%, median 6, IQR 1–20).

The composite nutritional phenotype distribution was as follows: 13,531 patients (50%) adequately nourished, 8,084 (30%) obese-replete, 1,597 (5.9%) sarcopenic-obese, and 3,845 (14%) cachectic-malnourished. Baseline characteristics by phenotype are presented in Supplementary Table 1. Patients in the sarcopenic-obese and cachectic-malnourished groups were older (median age 64 vs. 60 years for adequately nourished, *p* < 0.001), more likely to harbor brain metastases (36% and 53% vs. 31%, *p* < 0.001), and carried a higher frailty burden (median RAI 6 and 11 vs. 6, *p* < 0.001). Hypoalbuminemia was present in 83% of cachectic patients, and preoperative weight loss greater than 10% of body weight occurred almost exclusively in the cachectic group (11% vs. ≤ 1.2% in other phenotypes). Within the cachectic-malnourished group, 3,173 patients (82.5%) had hypoalbuminemia and 420 (10.9%) had documented preoperative weight loss greater than 10%, whereas the remaining 672 (17.5%) were classified as cachectic on the basis of low body mass index (< 18.5) or weight loss without hypoalbuminemia, indicating that the category predominantly reflects hypoalbuminemia rather than clinically defined cachexia. Chronic preoperative corticosteroid use was reported in 18% of the analytic cohort and was most prevalent in the cachectic group (23%). ASA classification ≥ 3 was substantially more frequent in the sarcopenic-obese (93%) and cachectic (91%) groups than in adequately nourished patients (80%).

### Major 30-day morbidity

**Fig. 1:**
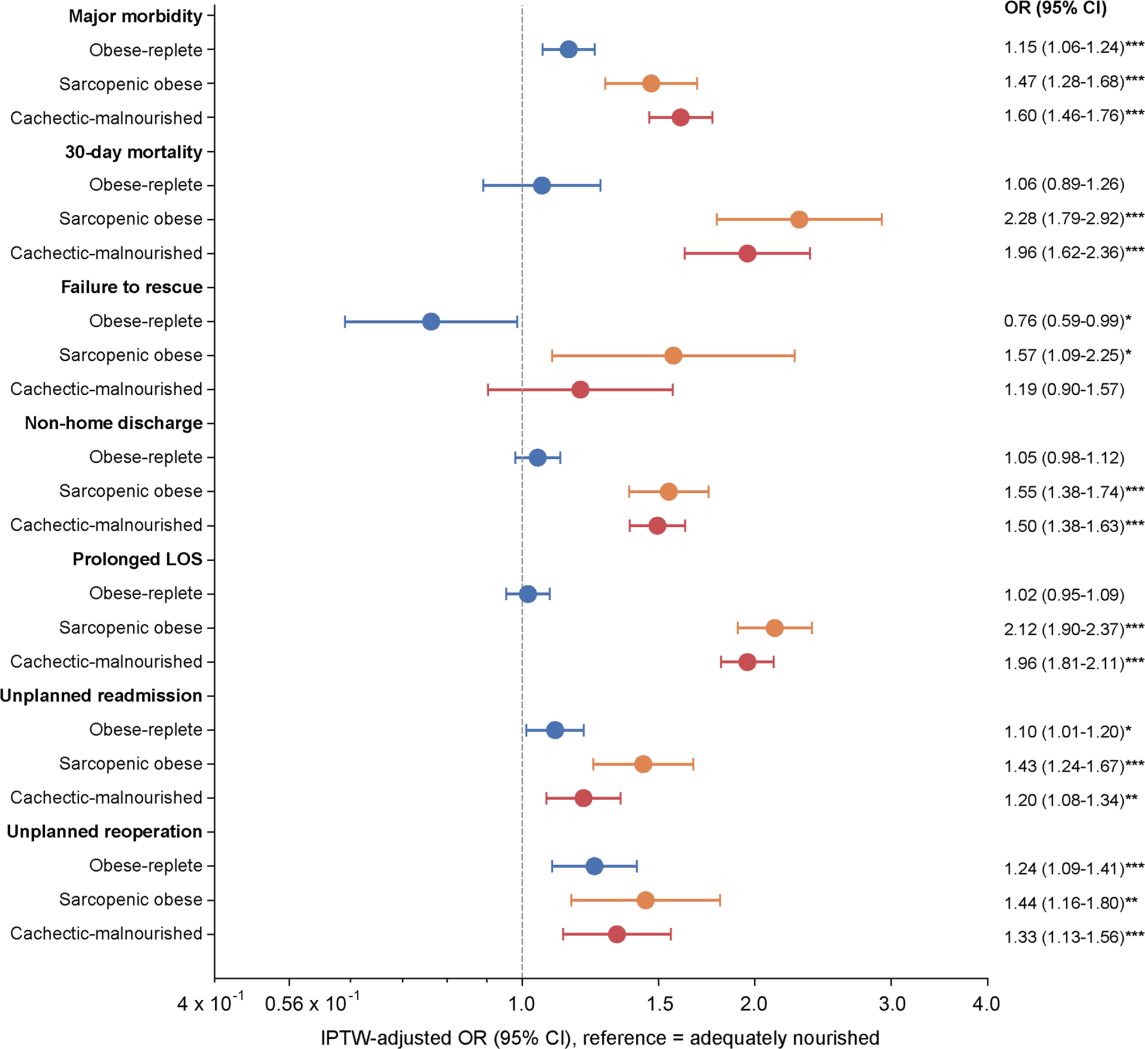
IPTW-adjusted associations between composite nutritional phenotype and 30-day outcomes. Forest plot of inverse probability of treatment weighting-adjusted odds ratios with 95% confidence intervals for each phenotype relative to the adequately nourished reference (vertical dashed line at IPTW OR = 1.0). Estimates are derived from generalized linear models with stabilized IPTW weights and robust standard errors (HC1). The composite phenotype was constructed a priori from BMI and serum albumin as detailed in the Methods. *p < 0.05; **p < 0.01; ***p < 0.001; † p < 0.10. ^1^or = odds ratio; ci = confidence interval; iptw = inverse probability of treatment weighting; los = length of stay; bmi = body mass index

Major 30-day morbidity occurred in 4,233 patients (16%). Compared with the adequately nourished reference, all three non-reference phenotypes were associated with elevated odds: obese-replete (IPTW OR 1.15, 95% CI 1.06–1.24, *p* < 0.001), sarcopenic-obese (IPTW OR 1.47, 95% CI 1.28–1.68, *p* < 0.001), and cachectic-malnourished (IPTW OR 1.60, 95% CI 1.46–1.76, *p* < 0.001) (Table 1; Fig. 1). The magnitude of association was greatest among cachectic patients and lowest among obese-replete patients.

### 30-day mortality

Thirty-day mortality occurred in 871 patients (3.2%). Unadjusted mortality was 2.2% in adequately nourished and 2.1% in obese-replete patients but rose to 6.9% in sarcopenic-obese and 7.7% in cachectic patients (Table 1). After IPTW adjustment, sarcopenic-obese (IPTW OR 2.28, 95% CI 1.79–2.92, *p* < 0.001) and cachectic (IPTW OR 1.96, 95% CI 1.62–2.36, *p* < 0.001) phenotypes carried substantially elevated mortality, while obese-replete patients did not differ from the adequately nourished reference (IPTW OR 1.06, 95% CI 0.89–1.26, *p* = 0.508) (Fig. 1).

### Failure to rescue

Among the 2,691 patients (9.9%) who experienced an FTR-qualifying complication, 462 (17%) died within 30 days. The obese-replete phenotype showed reduced odds of FTR (IPTW OR 0.76, 95% CI 0.59–0.99, *p* = 0.038), the sarcopenic-obese phenotype showed elevated odds (IPTW OR 1.57, 95% CI 1.09–2.25, *p* = 0.014), and the cachectic phenotype did not differ from reference (IPTW OR 1.19, 95% CI 0.90–1.57, *p* = 0.217) (Table 1; Fig. 1).

### Non-home discharge and tertiary outcomes

Non-home discharge occurred in 6,223 patients (23%). Sarcopenic-obese (IPTW OR 1.55, 95% CI 1.38–1.74, *p* < 0.001) and cachectic (IPTW OR 1.50, 95% CI 1.38–1.63, *p* < 0.001) phenotypes conferred elevated odds, while the obese-replete phenotype did not differ (IPTW OR 1.05, 95% CI 0.98–1.12, *p* = 0.178). Across tertiary outcomes, sarcopenic-obese patients had the largest effects for prolonged LOS (IPTW OR 2.12, 95% CI 1.90–2.37, *p* < 0.001) and unplanned readmission (IPTW OR 1.43, 95% CI 1.24–1.67, *p* < 0.001); all three non-reference phenotypes showed elevated odds of unplanned reoperation (Table 1).

### Histology-stratified phenotype effects

**Fig. 2:**
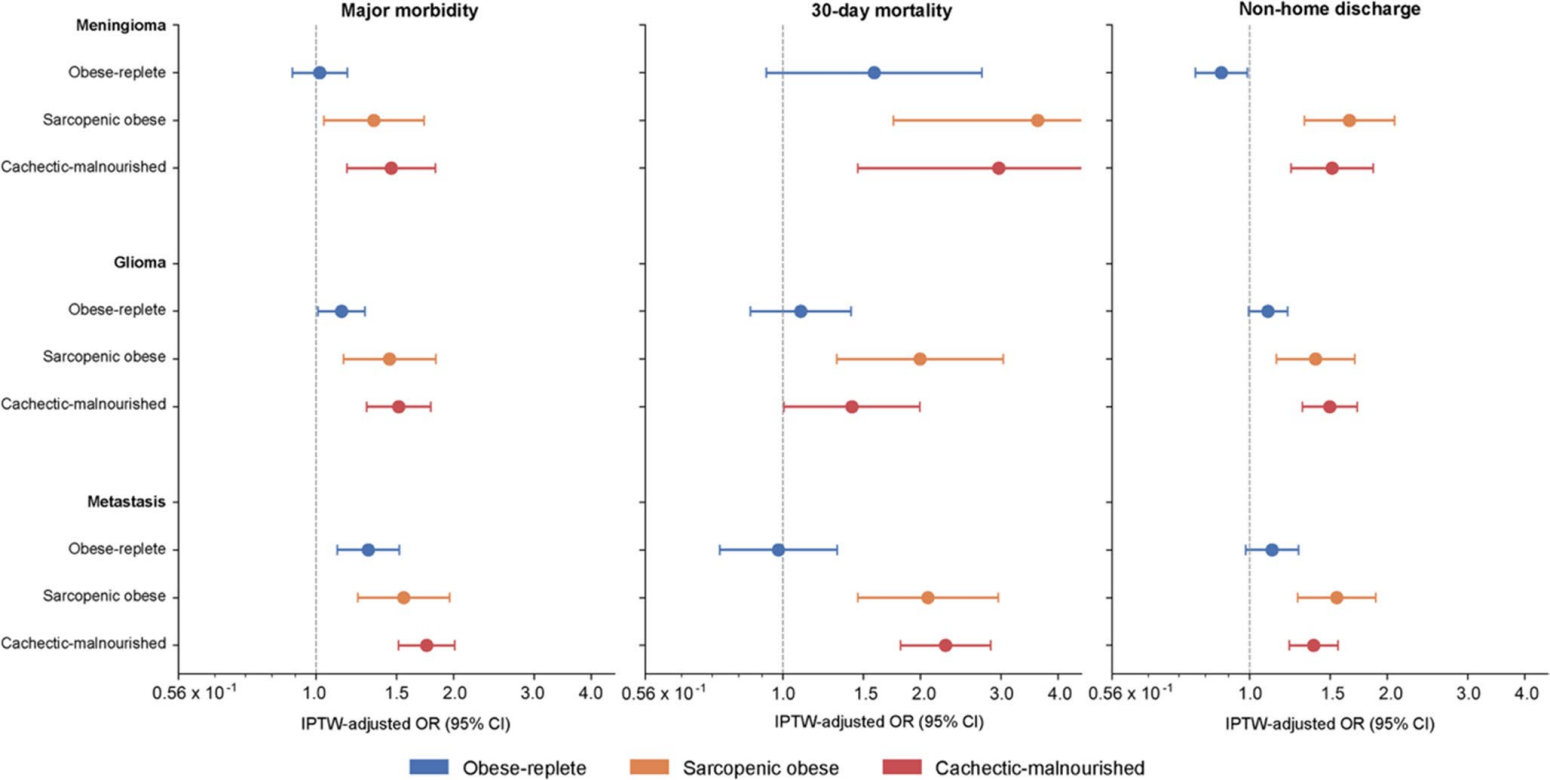
Histology-stratified phenotype-outcome associations. IPTW-adjusted odds ratios for major 30-day morbidity (left panel), 30-day mortality (middle panel), and non-home discharge (right panel) by tumor histology (meningioma, malignant glioma, metastasis). Propensity scores were re-estimated within each histologic stratum. The reference group within each stratum is adequately nourished. ^1^or = odds ratio; ci = confidence interval; iptw = inverse probability of treatment weighting

Phenotype-outcome associations differed across histologic strata. Formal interaction tests were significant for 30-day mortality (*p* = 0.044) and non-home discharge (*p* = 0.009) but not for major morbidity (*p* = 0.313) (Table 2; Fig. 2). In the meningioma cohort (*n* = 6,592; 88 deaths [1.3%]), the sarcopenic-obese phenotype carried the largest mortality estimate observed in any subgroup (IPTW OR 3.61, 95% CI 1.75–7.47, *p* < 0.001), followed by the cachectic phenotype (IPTW OR 2.97, 95% CI 1.46–6.05, *p* = 0.003). Because these subgroup estimates rest on few events (88 deaths across the meningioma stratum, among 449 sarcopenic-obese meningioma patients) and carry wide confidence intervals, they are hypothesis-generating. The obese-replete phenotype was associated with reduced odds of non-home discharge in meningioma patients (IPTW OR 0.87, 95% CI 0.76–0.99, *p* = 0.034). In the malignant glioma cohort (*n* = 11,873), a stepwise gradient of risk was observed across phenotypes for both major morbidity (obese-replete IPTW OR 1.14, sarcopenic-obese 1.45, cachectic 1.52; all *p* < 0.05) and non-home discharge (1.10, 1.39, 1.50). In the metastasis cohort (*n* = 8,592), phenotype effects on major morbidity were largest among the three histologies (1.30, 1.55, 1.75; all *p* < 0.001), and the obese-replete reduction in FTR was most pronounced (IPTW OR 0.58, 95% CI 0.36–0.91, *p* = 0.018).

**Table 2:** Histology-stratified IPTW-adjusted associations between composite nutritional phenotype and key 30-day outcomes

Outcome / phenotype	Meningioma OR (95% CI), *p*	Malignant glioma OR (95% CI), *p*	Metastasis OR (95% CI), *p*	*p*-value
Major 30-day morbidity				
Event rate, n (%)	1,214/6,592 (18%)	1,677/11,873 (14%)	1,342/8,592 (16%)	0.313
Obese-replete	1.02 (0.89-1.17), 0.800	1.14 (1.01-1.28), 0.033*	1.30 (1.11-1.52), <0.001***	
Sarcopenic-obese	1.34 (1.04-1.72), 0.024*	1.45 (1.15-1.83), 0.002**	1.55 (1.23-1.96), <0.001***	
Cachectic-malnourished	1.46 (1.17-1.83), <0.001***	1.52 (1.29-1.78), <0.001***	1.75 (1.52-2.01), <0.001***	
30-day mortality				
Event rate, n (%)	88/6,592 (1.3%)	333/11,873 (2.8%)	450/8,592 (5.2%)	0.044*
Obese-replete	1.58 (0.92-2.73), 0.097†	1.09 (0.85-1.41), 0.486	0.98 (0.73-1.32), 0.883	
Sarcopenic-obese	3.61 (1.75-7.47), <0.001***	2.00 (1.31-3.04), <0.001***	2.08 (1.46-2.96), <0.001***	
Cachectic-malnourished	2.97 (1.46-6.05), 0.003**	1.42 (1.01-1.99), 0.046	2.27 (1.81-2.85), <0.001***	
Failure to rescue				
Event rate, n (%)	68/662 (10%)	174/1,173 (15%)	220/856 (26%)	–
Obese-replete	1.11 (0.58-2.10), 0.756	0.81 (0.55-1.18), 0.273	0.58 (0.36-0.91), 0.018*	
Sarcopenic-obese	2.67 (1.19-5.98), 0.017*	1.59 (0.86-2.96), 0.139	1.09 (0.61-1.96), 0.761	
Cachectic-malnourished	1.68 (0.73-3.88), 0.226	1.00 (0.61-1.64), 0.995	1.17 (0.81-1.68), 0.402	
Non-home discharge				
Event rate, n (%)	1,435/6,592 (22%)	2,712/11,873 (23%)	2,076/8,592 (24%)	0.009**
Obese-replete	0.87 (0.76-0.99), 0.034*	1.10 (0.99-1.21), 0.065†	1.12 (0.98-1.28), 0.095†	
Sarcopenic-obese	1.65 (1.32-2.08), <0.001***	1.39 (1.14-1.70), <0.001***	1.55 (1.28-1.89), <0.001***	
Cachectic-malnourished	1.52 (1.23-1.87), <0.001***	1.50 (1.31-1.72), <0.001***	1.38 (1.22-1.56), <0.001***	

IPTW models fit separately within each histologic stratum, with propensity scores re-estimated using all covariates except histology dummies. Interaction p values from likelihood ratio test comparing models with versus without phenotype × histology interaction terms (df = 6). *p < 0.05; **p < 0.01; ***p < 0.001; † p < 0.10

^1^*or* odds ratio, *ci* confidence interval, *iptw* inverse probability of treatment weighting

### Sensitivity analyses

**Fig. 3:**
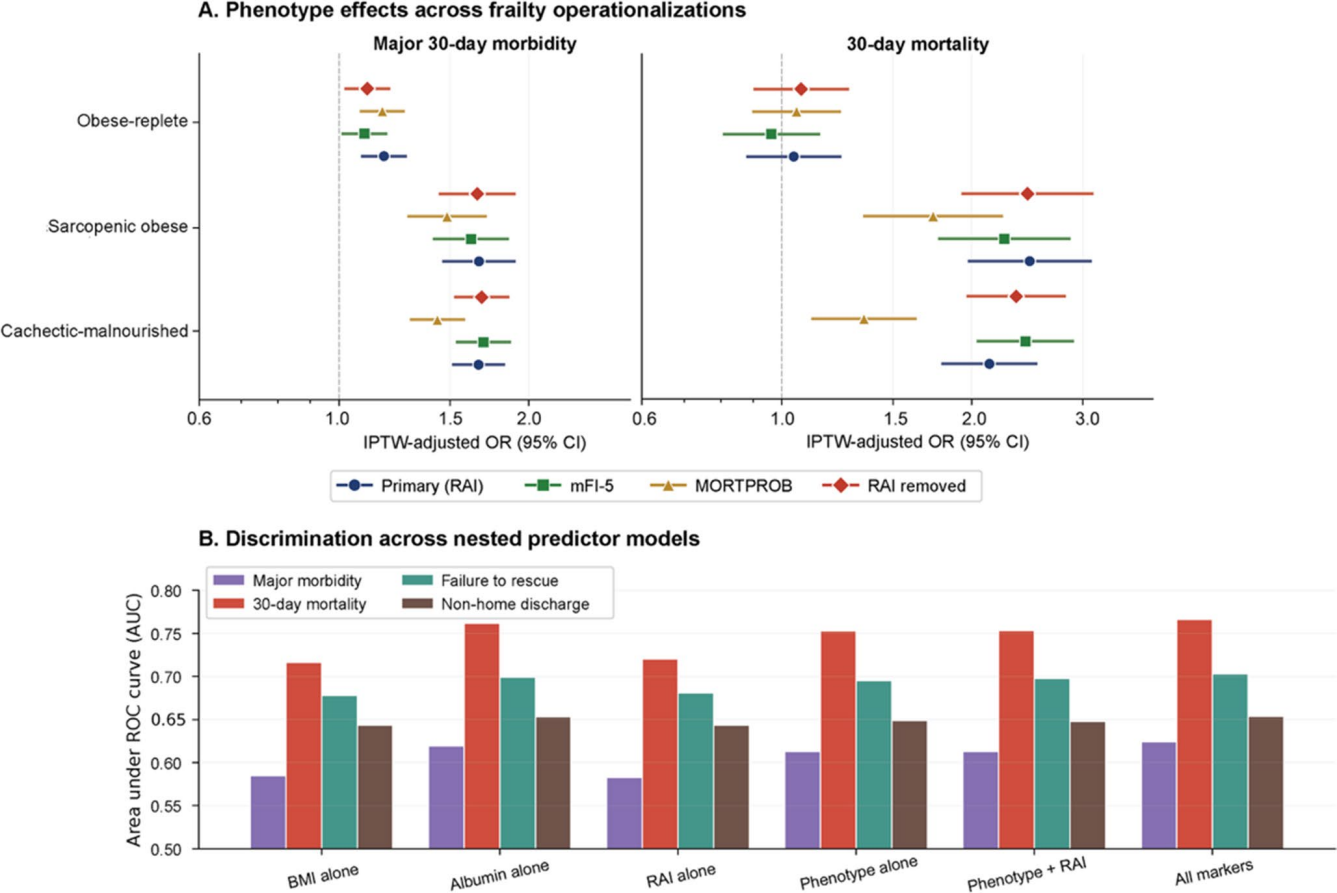
Phenotype effects across frailty operationalizations and discrimination across nested predictor models. (**A**) IPTW-adjusted odds ratios with 95% confidence intervals for each phenotype relative to the adequately nourished reference, computed under four frailty operationalizations: primary specification (RAI), mFI-5 substitution, ACS-NSQIP MORTPROB substitution, and RAI removed entirely. The left sub-plot shows major 30-day morbidity; the right sub-plot shows 30-day mortality. Estimates cluster tightly across specifications, indicating that phenotype-outcome associations are robust to alternative frailty operationalizations. (**B**) Area under the receiver operating characteristic curve (AUC) for nested logistic regression models containing different combinations of nutritional and frailty markers, evaluated across all four 30-day outcomes. All models additionally include age, age squared, sex, race/ethnicity, and tumor histology ^¹^or = odds ratio; ci = confidence interval; iptw = inverse probability of treatment weighting; rai = risk analysis index; mfi-5 = 5-item modified frailty index; mortprob = 30-day mortality probability from acs-nsqip universal surgical risk calculator; auc = area under the curve

Primary findings were robust across four sensitivity analyses (Supplementary Table 2). Multiple imputation on the 47,019-patient cohort yielded estimates in close agreement with the complete-case primary analysis (sarcopenic-obese major morbidity IPTW OR 1.57 vs. 1.47; cachectic 1.67 vs. 1.60). Replacing RAI with mFI-5 yielded estimates within 5% of the primary analysis across all phenotype-outcome pairings. Restricting the cohort to elective cases (*n* = 26,347) preserved both magnitude and significance of all primary associations. Substituting the ACS-NSQIP universal surgical risk calculator-derived MORTPROB for RAI as the frailty covariate modestly attenuated estimates but preserved directionality and significance for all primary outcomes (sarcopenic-obese mortality IPTW OR 1.85, 95% CI 1.44–2.37; cachectic 1.34, 95% CI 1.10–1.63). Estimates across all alternative frailty operationalizations are visualized in Fig. 3A.

### Discrimination of the composite phenotype versus single markers

Discrimination of nested predictive models was assessed across alternative covariate specifications (Fig. 3B, Supplementary Table 3). The fully specified model achieved the highest AUC across all four outcomes: 30-day mortality (0.77), major 30-day morbidity (0.62), FTR (0.70), and non-home discharge (0.65). The composite phenotype alone closely approached this performance for each outcome (0.75, 0.61, 0.70, and 0.65, respectively) but did not exceed serum albumin, the strongest single-marker predictor (0.76, 0.62, 0.70, and 0.65). A covariates-only model containing no nutritional or frailty marker (age, sex, race/ethnicity, and histology) achieved AUCs of 0.71 for mortality, 0.56 for major morbidity, and 0.66 for non-home discharge, so serum albumin accounted for most of the achievable discrimination from nutritional status, with the composite phenotype and albumin contributing comparably; internally validated (bootstrap) estimates are reported in Supplementary Table 3. BMI alone and RAI alone trailed across all outcomes (mortality each 0.72; morbidity 0.59 and 0.58; FTR each 0.68; non-home discharge each 0.64), and adding RAI to the phenotype yielded no incremental discrimination.

## Discussion

Patients undergoing ITR represent a heterogeneous population in whom short-term risk is shaped by tumor histology, systemic cancer burden, neurologic reserve, and baseline host physiology [27, 28]. Prior NSQIP neuro-oncology studies have shown that BMI and serum albumin carry prognostic information, but with divergent outcome profiles, suggesting each marker captures nonredundant dimensions of perioperative vulnerability. Dasenbrock et al. [2] found that obesity in 11,510 craniotomy patients was not associated with 30-day mortality or nonroutine discharge despite increased major complications, whereas hypoalbuminemia was associated with markedly higher 30-day mortality (9.6% vs. 2.9%) and nonroutine discharge. More recent NSQIP data from Varela et al. [24] confirmed that hypoalbuminemia independently predicts Clavien IV complications, reoperation, readmission, extended length of stay, and nonroutine discharge, although not 30-day mortality after adjustment. The present analysis builds upon these divergent patterns by integrating BMI and albumin into a four-category composite phenotype, resolving mechanistically distinct ITR risk groups beyond single markers. Serum albumin in this population reflects systemic inflammation, tumor burden, hepatic synthetic function, and corticosteroid exposure at least as much as nutritional intake, none of which is directly captured in ACS-NSQIP, so associations attributed to the nutritional phenotype should not be read as purely nutritional.

Sarcopenic-obese patients represented only 5.9% of the cohort but exhibited the highest risks across nearly every outcome, with greater odds of major morbidity and more than two-fold higher odds of mortality. This phenotype combines obesity-related metabolic dysfunction with protein depletion, resulting in elevated physiologic stress and reduced anabolic reserve [29]. While this construct aligns with the 2022 ESPEN/EASO consensus [15] and is supported by prior demonstrations in non-cranial oncologic surgery [30], it has not yet been operationalized as a preoperative risk category in intracranial tumor cohorts. It is clinically invisible within BMI-based stratification and conflated with cachexia under albumin-based stratification despite distinct underlying physiology [31, 32]. In contrast, the cachectic-malnourished phenotype exhibited the highest absolute mortality without a significant FTR signal, likely reflecting the underlying oncologic trajectory of the 53% of cachectic patients harboring brain metastases [14]. The obese-replete phenotype was associated with reduced FTR, most pronounced in the metastases subgroup. This parallels the obesity paradox documented in oncologic surgical populations [6] and demonstrates the protective effect of adipose reserves on recovery from severe complications [33]. Importantly, this protection depends on nutritional adequacy, as obese patients with hypoalbuminemia in our cohort carried the worst overall risk profile.

Sarcopenic obesity was associated with a 3.61-fold increase in 30-day mortality for meningioma patients; a magnitude approaching the risk seen after malignant ITR, despite meningiomas typically representing a lower-mortality, extra-axial neuro-oncologic population [34, 35]. Parallel studies have revealed meningioma-specific risk patterns: Khazanchi et al. [36] showed that obesity in this population independently increases the risk of postoperative pulmonary embolism, and Dickinson et al. [37] found that preoperative weight loss is strongly associated with increased mortality after meningioma craniotomy. Meningioma risk stratification typically involves tumor size, location, peritumoral edema, venous sinus involvement, and global frailty [26, 38], but sarcopenic obesity is not yet considered a distinct preoperative phenotype. This sarcopenic-obese phenotype represents a high-risk and clinically occult vulnerability state. Further work is needed to clarify the mechanisms underlying this mortality signal and to determine whether prospective identification of sarcopenic obesity should inform perioperative optimization and meningioma-specific management guidelines [25]. Conversely, the reduced FTR associated with the obese-replete phenotype was most pronounced in the brain metastasis cohort, where systemic disease burden, baseline nutritional reserve, and tolerance of postoperative complications are particularly relevant [39]. These findings demonstrate that nutritional phenotype effects vary meaningfully across intracranial tumor histologies and perioperative endpoints.

Several methodological choices distinguish this analysis from prior NSQIP studies. Phenotypes were defined a priori using validated international consensus criteria [14, 15, 40] to protect against post hoc category optimization. Histology stratification with formal interaction testing permitted direct quantification of effect modification rather than indirect inference [4, 5, 13]. IPTW with stabilized weights achieved post-weighting SMDs below 0.10 for all measured covariates; because a nutritional phenotype has no coherent counterfactual intervention, we interpret the weighted estimates as confounding-adjusted associations under standard exchangeability assumptions rather than average treatment effects. Sensitivity analysis with alternative frailty operationalizations preserved directionality and revealed that while frailty remains a clinically meaningful construct of physiologic reserve and an established predictor of FTR and mortality in intracranial tumor cohorts [5, 12, 13], BMI and nutritional status are the operative drivers of the observed risk gradient.

Different phenotypes may benefit from distinct preoperative strategies adapted from oncologic prehabilitation frameworks and tailored to intracranial tumor surgery [39, 41]. Sarcopenic-obese patients may benefit from protein-focused nutritional optimization and resistance-based prehabilitation rather than weight loss alone, cachectic patients from expedited nutritional repletion and systemic disease optimization, and obese-replete patients from standard perioperative pathways [42, 43]. These strategies require weeks of protein loading and resistance training to alter body composition, an interval unavailable to most patients presenting with symptomatic mass effect, so their applicability is largely limited to the subset undergoing elective, deferrable resection. Preoperative imaging may also allow more direct assessment of body composition: Morshed et al. [44] reported that decreased masseter diameter on preoperative MRI predicts shorter overall survival and 90-day mortality after GBM resection, and Khalid et al. [45] found that imaging-derived sarcopenic obesity on preoperative CT predicted non-home discharge, readmission, and 90-day and 1-year mortality after spinal metastasis surgery. These findings support future imaging-derived operationalization of sarcopenic obesity using direct measures of muscle and adiposity not captured in NSQIP [21, 46].

This study has several strengths. The analytic cohort of 27,057 ITRs from 2016 to 2023 extends upon the temporal coverage of prior analyses [12, 13] into the post-2020 era. Comparative discrimination analyses showed that albumin was the strongest single-marker predictor of 30-day mortality, while RAI offered no advantage over BMI alone. The composite phenotype achieved discrimination comparable to serum albumin alone, while providing a clinically interpretable framework that distinguishes mechanistically distinct high-risk subgroups that single biomarkers cannot identify. Histology stratification with formal interaction testing revealed clinically important effect modification with direct implications for risk communication and patient selection. Sensitivity analyses across alternative frailty operationalizations and missingness handling yielded estimates within clinically negligible margins of the primary findings.

Several limitations warrant consideration. The phenotype categories are administrative surrogates rather than validated clinical diagnoses: serum albumin is not a component of any consensus definition of sarcopenic obesity, which requires direct measurement of muscle mass or function, so the sarcopenic-obese label identifies obese patients with hypoalbuminemia rather than confirmed sarcopenia, and only 11% of the cachectic-malnourished group had documented weight loss exceeding 10%, so that category predominantly reflects isolated hypoalbuminemia and also includes underweight patients with preserved albumin who may not meet clinical criteria for cachexia. The analysis is restricted to 30-day perioperative outcomes within ACS-NSQIP and does not address long-term oncologic outcomes or functional status beyond discharge. Although IPTW achieved measured covariate balance, residual confounding from unmeasured tumor characteristics cannot be excluded. FTR analyses were limited to patients with qualifying complications (*n* = 2,691), reducing precision in histology-stratified estimates. Finally, primary analyses were restricted to the 58% of eligible cases with complete BMI and albumin data. Because serum albumin is ordered selectively rather than universally in ACS-NSQIP, these data are unlikely to be missing completely at random, and multiple imputation assumes rather than tests the missing-at-random condition; the agreement between imputed and complete-case estimates therefore reflects robustness to the imputation model rather than confirmation that data were missing at random, and informative missingness driven by unmeasured illness severity cannot be excluded.

## Conclusion

In a histology-stratified analysis of 27,057 ITRs, a biomarker-defined nutritional phenotype constructed from BMI and serum albumin separated clinically distinct high-risk subgroups but did not improve discrimination beyond serum albumin alone. Sarcopenic-obese and cachectic-malnourished phenotypes were associated with elevated odds of major morbidity and 30-day mortality, and phenotype effects varied by histology. The elevated mortality observed among sarcopenic-obese meningioma patients rests on few events and requires prospective confirmation. These findings support histology-aware nutritional phenotyping for preoperative risk communication and hypothesis generation rather than as a substitute for single-marker assessment.

## Supplementary information

Below is the link to the electronic supplementary material.

Supplementary Material 1 (DOCX 184 KB)

## Data Availability

The data that support the findings of this study were obtained from the American College of Surgeons National Surgical Quality Improvement Program (ACS-NSQIP) Participant Use Data File. Restrictions apply to the availability of these data, which were used under a Participant Use File Data Use Agreement and are therefore not publicly available. Qualified researchers may obtain access through application to the American College of Surgeons. Analytic code is available from the corresponding author upon reasonable request.
