# Supplementary material for: Sarcopenic obesity and cachexia as nutritional risk phenotypes and histology-specific outcomes after intracranial tumor resection: a histology-stratified NSQIP analysis of 27,057 cases
Source: Neurosurg Rev. 2026 Aug 18;49(1):525. doi: 10.1007/s10143-026-04450-3 (PMC13486080; doi:10.1007/s10143-026-04450-3)
Supplement: Supplementary file 1 [file 10143_2026_4450_MOESM1_ESM.docx]

**Neurosurgical Review**

**Title:** Sarcopenic Obesity and Cachexia as Distinct Risk Phenotypes After Intracranial Tumor Resection: A Histology-Stratified NSQIP Analysis of 27,057 Cases

**Authors:** Caleigh S. Roach, BA^1^; Belen Wertheimer, BA^2^; Khushi H. Shah, BS^1^; Victor M. Lu, MD, PhD^1^; Ashish H. Shah, MD^1^; Ricardo J. Komotar, MD^1^

**Affiliations:**

^1^Department of Neurological Surgery, University of Miami Miller School of Medicine, Miami, FL, USA

^2^Department of Orthopaedic Surgery, University of Miami Miller School of Medicine, Miami, FL, USA

**Corresponding Author:**

Caleigh S. Roach, BA

Department of Neurological Surgery, University of Miami Miller School of Medicine

1295 NW 14th Street, Suite 1700

Miami, FL, USA, 33125

caleighroach@med.miami.edu

https://orcid.org/0009-0005-1577-2112

**Supplementary Data**

**Supplementary Fig. 1 Cohort selection flow diagram.** Sequential application of inclusion and exclusion criteria from the ACS-NSQIP Participant Use File (2016-2023) to the primary analytic cohort. The four-category composite nutritional phenotype was assigned a priori using preoperative BMI, serum albumin, and documented preoperative weight loss as defined in the Methods. Histologic distribution within the primary cohort is shown in the lower panel.

*^1^ acs-nsqip = american college of surgeons national surgical quality improvement program; cpt = current procedural terminology; or = operating room; bmi = body mass index.*

**Supplementary Table 1. Baseline demographic, clinical, and operative characteristics by composite nutritional phenotype.**

Values are n (%) for categorical variables, median (IQR) or mean (SD) for continuous variables as indicated. P values from chi-squared test for categorical variables, ANOVA for normally distributed continuous variables, and Kruskal-Wallis test otherwise. *p < 0.05; **p < 0.01; ***p < 0.001; † p < 0.10.

*^1^ bmi = body mass index; iqr = interquartile range; sd = standard deviation; rai = risk analysis index; mfi-5 = 5-item modified frailty index; asa = American Society of Anesthesiologists; copd = chronic obstructive pulmonary disease; chf = congestive heart failure; wbc = white blood cell count; inr = international normalized ratio.*

| Characteristic | Overall | Adequately nourished | Obese-replete | Sarcopenic-obese | Cachectic-malnourished | *p*-value |
| --- | --- | --- | --- | --- | --- | --- |
| No. of patients | 27,057 | 13,531 | 8,084 | 1,597 | 3,845 | - |
| Demographics |  |  |  |  |  |  |
| Age, years, median (IQR) | 61 (50-69) | 60 (49-69) | 59 (48-67) | 64 (55-71) | 64 (56-72) | <0.001*** |
| <50, n (%) | 6,540 (24%) | 3,536 (26.1) | 2,217 (27%) | 238 (15%) | 549 (14%) |  |
| 50-64, n (%) | 10,124 (37%) | 4,852 (36%) | 3,301 (41%) | 587 (37%) | 1,384 (36%) |  |
| 65-74, n (%) | 7,041 (26%) | 3,429 (25%) | 1,886 (23%) | 513 (32%) | 1,213 (32%) |  |
| ≥75, n (%) | 3,352 (12%) | 1,714 (13%) | 680 (8.4%) | 259 (16%) | 699 (18%) |  |
| Female sex, n (%) | 13,832 (51%) | 6,697 (50%) | 4,358 (54%) | 895 (56%) | 1,882 (49%) | <0.001*** |
| Race/ethnicity, n (%) |  |  |  |  |  | <0.001*** |
| White, non-Hispanic | 16,954 (63%) | 8,592 (64%) | 5,219 (65%) | 946 (59%) | 2,197 (57%) |  |
| Black, non-Hispanic | 2,154 (8.0%) | 812 (6.0%) | 717 (8.9) | 210 (13%) | 415 (11%) |  |
| Hispanic | 2,016 (7.5%) | 932 (6.9%) | 659 (8.2) | 147 (9.2%) | 278 (7.2%) |  |
| Asian | 945 (3.5%) | 645 (4.8%) | 103 (1.3) | 17 (1.1%) | 180 (4.7%) |  |
| Other/Unknown | 4,988 (18%) | 2,550 (19%) | 1,386 (17.1) | 277 (17%) | 775 (20%) |  |
| Tumor histology, n (%) |  |  |  |  |  | <0.001*** |
| Meningioma | 6,592 (24%) | 2,963 (22%) | 2,632 (33%) | 449 (28%) | 548 (14%) |  |
| Malignant glioma | 11,873 (44%) | 6,379 (47%) | 3,644 (45%) | 577 (36%) | 1,273 (33%) |  |
| Metastasis | 8,592 (32%) | 4,189 (31%) | 1,808 (22%) | 571 (36%) | 2,024 (53%) |  |
| Tumor location, n (%) |  |  |  |  |  | <0.001*** |
| Supratentorial | 23,240 (86%) | 11,637 (86.0) | 7,084 (87.6) | 1,376 (86.2) | 3,143 (81.7) |  |
| Infratentorial | 3,752 (14%) | 1,859 (13.7) | 980 (12.1) | 218 (13.7) | 695 (18.1) |  |
| Other skull base | 65 (0.2%) | 35 (0.3) | 20 (0.2) | 3 (0.2) | 7 (0.2) |  |
| Body composition and nutrition |  |  |  |  |  |  |
| BMI, kg/m², mean (SD) | 28.7 (6.6%) | 25.2 (2.9) | 35.5 (5.2) | 36.3 (5.9) | 23.6 (4.0) | <0.001*** |
| Albumin, g/dL, mean (SD) | 3.9 (0.5%) | 4.1 (0.4) | 4.1 (0.4) | 3.1 (0.3) | 3.2 (0.5) | <0.001*** |
| Hypoalbuminemia (<3.5 g/dL), n (%) | 4,770 (18%) | 0 (0.0) | 0 (0.0) | 1,597 (100.0) | 3,173 (82.5) | 0 |
| >10% weight loss in 6 mo, n (%) | 479 (1.8%) | 0 (0.0) | 40 (0.5) | 19 (1.2) | 420 (10.9) | 0 |
| Frailty |  |  |  |  |  |  |
| RAI score, median (IQR) | 6 (1-20) | 6 (1-20) | 6 (1-9) | 6 (1-21) | 11 (5-24) | <0.001*** |
| RAI tier, n (%) |  |  |  |  |  | <0.001*** |
| Robust | 21,068 (78%) | 10,548 (78.0) | 6,792 (84%) | 1,179 (74%) | 2,549 (66%) |  |
| Normal | 5,452 (20%) | 2,796 (21%) | 1,198 (15%) | 368 (23%) | 1,090 (28%) |  |
| Frail | 494 (1.8%) | 170 (1.3%) | 91 (1.1%) | 46 (2.9%) | 187 (4.9%) |  |
| Severely frail | 43 (0.2%) | 17 (0.1%) | 3 (0.0%) | 4 (0.3%) | 19 (0.5%) |  |
| mFI-5, mean (SD) | 0.1 (0.2%) | 0.1 (0.1%) | 0.2 (0.2%) | 0.2 (0.2%) | 0.2 (0.2%) | <0.001*** |
| Functional dependence, n (%) | 1,280 (4.7%) | 483 (3.6%) | 313 (3.9%) | 143 (9.0%) | 341 (8.9%) | <0.001*** |
| Comorbidities, n (%) |  |  |  |  |  |  |
| Hypertension | 11,333 (42%) | 4,676 (35%) | 4,024 (49.8) | 969 (60.7) | 1,664 (43.3) | <0.001*** |
| Diabetes mellitus | 4,137 (15%) | 1,460 (11%) | 1,652 (20.4) | 454 (28.4) | 571 (14.9) | <0.001*** |
| Current smoker | 4,647 (17%) | 2,289 (17%) | 1,015 (12.6) | 250 (15.7) | 1,093 (28.4) | <0.001*** |
| COPD | 1,356 (5.0%) | 560 (4.1%) | 300 (3.7) | 118 (7.4) | 378 (9.8) | <0.001*** |
| CHF | 323 (1.2%) | 115 (0.8%) | 97 (1.2) | 43 (2.7) | 68 (1.8) | <0.001*** |
| Dialysis | 71 (0.3%) | 20 (0.1%) | 11 (0.1) | 15 (0.9) | 25 (0.7) | <0.001*** |
| Disseminated cancer | 8,279 (31%) | 4,001 (30%) | 1,762 (21.8) | 555 (34.8) | 1,961 (51.0) | <0.001*** |
| Chronic steroid use | 4,853 (18%) | 2,301 (17%) | 1,323 (16.4) | 347 (21.7) | 882 (22.9) | <0.001*** |
| Bleeding disorder | 787 (2.9%) | 315 (2.3%) | 208 (2.6) | 80 (5.0) | 184 (4.8) | <0.001*** |
| Operative characteristics |  |  |  |  |  |  |
| ASA classification, n (%) |  |  |  |  |  | <0.001*** |
| Class 1 | 136 (0.5%) | 97 (0.7%) | 24 (0.3%) | 0 (0.0) | 15 (0.4) |  |
| Class 2 | 4,222 (16%) | 2,570 (19%) | 1,209 (15%) | 104 (6.5%) | 339 (8.8%) |  |
| Class 3 | 18,378 (68%) | 9,044 (67%) | 5,746 (71%) | 1,091 (68%) | 2,497 (65%) |  |
| Class 4 | 4,195 (16%) | 1,763 (13%) | 1,071 (13%) | 386 (24%) | 975 (25%) |  |
| Class 5 | 71 (0.3) | 33 (0.2%) | 17 (0.2%) | 11 (0.7) | 10 (0.3%) |  |
| Emergent, n (%) | 710 (2.6%) | 362 (2.7%) | 170 (2.1%) | 44 (2.8) | 134 (3.5%) | <0.001*** |
| Inpatient setting, n (%) | 26,930 (100%) | 13,451 (99%) | 8,051 (100%) | 1,590 (100%) | 3,838 (100%) | 0.008** |
| Operative time, min, median (IQR) | 183 (130-257) | 182 (130-256) | 192 (135-271) | 182 (128-250) | 167 (121-232) | <0.001*** |
| Surgery 2020-2023 (vs 2016-2019), n (%) | 14,938 (55%) | 7,585 (56%) | 4,610 (57%) | 794 (50%) | 1,949 (51%) | <0.001*** |
| Preoperative labs, mean (SD) |  |  |  |  |  |  |
| Sodium, mEq/L | 138.5 (3.3) | 138.6 (3.2) | 138.8 (3.1) | 138.3 (3.4) | 137.8 (3.7) | <0.001*** |
| Creatinine, mg/dL | 0.9 (0.4) | 0.8 (0.3) | 0.9 (0.3) | 0.9 (0.7) | 0.8 (0.5) | <0.001*** |
| WBC, ×10³/µL | 9.8 (4.5) | 9.4 (4.4) | 9.8 (4.3) | 11.1 (4.6) | 10.9 (5.2) | <0.001*** |
| Hematocrit, % | 40.1 (5.0) | 40.4 (4.6) | 41.1 (4.6) | 38.4 (5.6) | 37.3 (5.6) | <0.001*** |
| Platelets, ×10³/µL | 248.9 (83.1) | 247.0 (78.5) | 250.3 (77.6) | 242.9 (89.1) | 255.2 (104.6) | <0.001*** |
| INR | 1.0 (0.2) | 1.0 (0.2) | 1.0 (0.2) | 1.1 (0.2) | 1.0 (0.1) | <0.001*** |

**Supplementary Table 2. Sensitivity analyses of phenotype-outcome associations across alternative analytic specifications.**

All estimates are IPTW-adjusted odds ratios (95% confidence intervals), reference category = adequately nourished. Multiple imputation was performed using iterative imputation by chained equations (5 imputations, BayesianRidge estimator) on the full 47,019-patient cohort using all baseline covariates and outcome variables as imputation predictors; estimates pooled using Rubin's rules. The mFI-5 substitution analysis replaced the Risk Analysis Index with the 5-item Modified Frailty Index as the frailty covariate in both the propensity score and outcome models. The MORTPROB substitution analysis replaced the Risk Analysis Index with the ACS-NSQIP universal surgical risk calculator-derived 30-day mortality probability, a validated multivariable risk metric universally captured in NSQIP. The elective-only analysis restricted the cohort to patients with non-emergent presentations.

*^1^ iptw = inverse probability of treatment weighting; rai = risk analysis index; mfi-5 = 5-item modified frailty index; mortprob = 30-day mortality probability from acs-nsqip universal surgical risk calculator.*

| Outcome / phenotype | Primary IPT  (n=27,057) | Multiple imputation (N=47,019) | mFI-5 substituted for RAI (n=27,057) | NSQIP MORTPROB substituted (n=27,057) | Elective only (n=26,347) |
| --- | --- | --- | --- | --- | --- |
| Major morbidity |  |  |  |  |  |
| Obese-replete | 1.15 (1.06-1.24) | 1.16 (1.09-1.23) | 1.15 (1.06-1.24) | 1.15 (1.06-1.24) | 1.15 (1.07-1.25) |
| Sarcopenic-obese | 1.47 (1.28-1.68) | 1.57 (1.38-1.78) | 1.46 (1.28-1.68) | 1.34 (1.17-1.54) | 1.48 (1.28-1.69) |
| Cachectic-malnourished | 1.60 (1.46-1.76) | 1.67 (1.54-1.82) | 1.60 (1.45-1.75) | 1.43 (1.30-1.57) | 1.60 (1.46-1.76) |
| 30-day mortality |  |  |  |  |  |
| Obese-replete | 1.06 (0.89-1.26) | 1.00 (0.87-1.16) | 1.06 (0.89-1.26) | 0.99 (0.84-1.16) | 1.11 (0.93-1.33) |
| Sarcopenic-obese | 2.28 (1.79-2.92) | 2.68 (2.12-3.38) | 2.27 (1.78-2.91) | 1.85 (1.44-2.37) | 2.39 (1.86-3.08) |
| Cachectic-malnourished | 1.96 (1.62-2.36) | 2.27 (1.92-2.69) | 1.95 (1.62-2.35) | 1.34 (1.10-1.63) | 1.99 (1.64-2.42) |
| Non-home discharge |  |  |  |  |  |
| Obese-replete | 1.05 (0.98-1.12) | - | 1.05 (0.98-1.12) | 1.05 (0.98-1.12) | 1.05 (0.98-1.12) |
| Sarcopenic-obese | 1.55 (1.38-1.74) | - | 1.55 (1.38-1.74) | 1.39 (1.23-1.57) | 1.57 (1.39-1.77) |
| Cachectic-malnourished | 1.50 (1.38-1.63) | - | 1.49 (1.38-1.62) | 1.38 (1.27-1.50) | 1.48 (1.36-1.62) |

**Supplementary Table 3. Discrimination across nested predictive models for the four primary 30-day outcomes.**

Area under the receiver operating characteristic curve (AUC) with 95% confidence intervals (bootstrap, 200 resamples) across nested model specifications for each of the four primary 30-day outcomes. A covariates-only reference model (age, age squared, sex, race/ethnicity, and histology, without any nutritional or frailty marker) is shown in the first data row to indicate the discrimination attributable to nutritional status; the failure-to-rescue covariates-only model was not estimated. All models include the covariates-only terms. Adding serum albumin or the composite phenotype improved discrimination over the covariates-only model, whereas the composite phenotype did not exceed serum albumin alone and RAI added no incremental discrimination.

*^1^ auc = area under the curve; ci = confidence interval; bmi = body mass index; rai = risk analysis index.*

| Model specification | Predictors included | Major morbidity | 30-day mortality | Failure to rescue | Non-home discharge |
| --- | --- | --- | --- | --- | --- |
| Covariates only | - Age, age squared, sex, race/ethnicity, histology (no nutritional or frailty marker) | 0.56 (0.56-0.57) | 0.71 (0.70-0.73) | not estimated | 0.66 (0.65-0.66) |
| BMI alone | - BMI (kg/m2) | 0.59 (0.58-0.59) | 0.72 (0.70-0.73) | 0.68 (0.65-0.70) | 0.64 (0.63-0.65) |
| Albumin alone | - Serum albumin (g/dL) | 0.62 (0.61-0.63) | 0.76 (0.75-0.78) | 0.70 (0.67-0.73) | 0.65 (0.65-0.66) |
| RAI alone | - RAI score (0-81 points) | 0.58 (0.57-0.59) | 0.72 (0.70-0.73) | 0.68 (0.66-0.71) | 0.64 (0.63-0.65) |
| Phenotype alone | - Four-category composite phenotype | 0.61 (0.60-0.62) | 0.75 (0.74-0.77) | 0.70 (0.67-0.72) | 0.65 (0.64-0.66) |
| Phenotype + RAI | - Four-category composite phenotype  - RAI (0-81 points) | 0.61 (0.60-0.62) | 0.75 (0.74-0.77) | 0.70 (0.67-0.72) | 0.65 (0.64-0.66) |
| All markers | - Four-category composite phenotype  - BMI (kg/m2)  - Serum albumin (g/dL)  - RAI score (0-81 points) | 0.62 (0.62-0.63) | 0.77 (0.75-0.78) | 0.70 (0.68-0.73) | 0.65 (0.65-0.66) |

**Supplementary Methods**

***Cohort selection and histology assignment***

Adult patients (aged 18 years or older) undergoing intracranial tumor resection were identified from the ACS-NSQIP Participant Use File (2016-2023) using the following primary Current Procedural Terminology (CPT) codes: 61510, 61512, 61518, 61519, 61520, 61521, 61526, 61530, 61545, 61546, 61548, 61601, 61608, 61615, and 62165. Histology was assigned using the postoperative principal ICD-10 diagnosis code (PODIAG10) where available, with CPT codes used as a fallback, according to the following mutually exclusive algorithm: meningioma, ICD-10 D32.x, D42.0, or C70.0, or CPT 61512 or 61519; malignant primary tumor (glioma or glioblastoma), ICD-10 C71.x; and brain metastasis, ICD-10 C79.3x. Cases were excluded for non-neurosurgical operating-surgeon specialty, an unassignable histologic category, operative time below 60 minutes, or missing both serum albumin and body mass index. Codes are summarized in Supplementary Table 4.

***Failure-to-rescue definition***

Failure to rescue was defined as 30-day mortality following one or more prespecified serious complications: unplanned reintubation, ventilator dependence beyond 48 hours, pulmonary embolism, deep venous thrombosis, cardiac arrest, myocardial infarction, cerebrovascular accident, acute renal failure or progressive renal insufficiency, sepsis or septic shock, or organ-space surgical site infection. Pneumonia, superficial and deep surgical site infection, urinary tract infection, bleeding requiring transfusion, and wound dehiscence were not counted as failure-to-rescue-qualifying complications. The denominator was restricted to the 2,691 patients in the primary analytic cohort who experienced at least one qualifying complication, a deliberately narrower set than the broader major-morbidity composite (n=4,233).

***Risk Analysis Index calculation***

The Revised Risk Analysis Index (RAI-rev) was computed as a weighted sum across domains following the Hall et al. (JAMA Surgery 2017) specification. Age contributions were weighted by cancer status, with patients harboring glioma or brain metastasis, or with a positive disseminated-cancer flag, receiving cancer-modified age weights (ages 65-69, 20 points; 70-74, 19; 75-79, 18; 80-84, 17; and 85 or older, 16), with an additional 1 point for cancer presence; patients without cancer received unmodified age weights of 2, 3, 4, 5, and 6 points, respectively. Other domain weights were male sex (+5), end-stage renal disease or current dialysis (+6), congestive heart failure (+4), dyspnea at rest or with moderate exertion (+8), preoperative weight loss greater than 10% of body weight within 6 months (+5), residence at a nursing home or chronic-care facility (+8), partial functional dependence (+8), and total functional dependence (+16). Poor appetite is not a discrete NSQIP variable and was omitted; the underlying construct of chronic undernutrition is captured through the composite phenotype via serum albumin and weight loss.

***Inverse probability of treatment weighting***

A multinomial logistic regression model was fit with the four-level composite phenotype as the categorical outcome and all Methods covariates as predictors. Predicted probabilities of phenotype membership conditional on covariates were extracted, and stabilized weights were computed as the marginal probability of phenotype membership divided by the conditional probability. Weights were truncated at the 1st and 99th percentiles. Covariate balance after weighting was assessed by standardized mean differences across phenotype pairs; all weighted standardized mean differences were below the 0.10 threshold. Because a nutritional phenotype has no coherent counterfactual intervention, weighted estimates are interpreted as confounding-adjusted associations rather than average treatment effects.

**Supplementary Table 4. CPT and ICD-10 codes used for cohort selection and histology assignment.**

*^1^ cpt = current procedural terminology; icd-10 = international classification of diseases, 10th revision; bmi = body mass index.*

| **Component** | **Codes / definition** |
| --- | --- |
| Inclusion CPT (craniotomy/craniectomy for tumor and skull base approaches) | 61510, 61512, 61518, 61519, 61520, 61521, 61526, 61530, 61545, 61546, 61548, 61601, 61608, 61615, 62165 |
| Meningioma | ICD-10 D32.x, D42.0, or C70.0 (or CPT 61512 or 61519 as fallback) |
| Malignant primary (glioma/glioblastoma) | ICD-10 C71.x |
| Brain metastasis | ICD-10 C79.3x |
| Histology source | Postoperative principal ICD-10 diagnosis (PODIAG10) preferred; CPT fallback |
| Exclusions | Non-neurosurgical operating-surgeon specialty; unassignable histologic category; operative time <60 min; missing both serum albumin and BMI |
